# Relationship between Serum 25-Hydroxyvitamin D Levels and Hormonal Status in Infertile Women: A Retrospective Study

**DOI:** 10.3390/diagnostics13193024

**Published:** 2023-09-22

**Authors:** Melinda Kolcsár, Bernadett Berecki, Zsolt Gáll

**Affiliations:** 1 Department of Pharmacology and Clinical Pharmacy, George Emil Palade University of Medicine, Pharmacy, Science and Technology of Targu Mures, Gheorghe Marinescu Street 38, 540142 Targu Mures, Romania; melinda.kolcsar@umfst.ro; 2 Faculty of Medicine, George Emil Palade University of Medicine, Pharmacy, Science and Technology of Targu Mures, Gheorghe Marinescu Street 38, 540142 Targu Mures, Romania; bernadett2007@yahoo.com

**Keywords:** infertility, vitamin D, anovulation, mid-luteal progesterone

## Abstract

(1) Background: Infertility is a prevalent issue that affects 10–18% of couples worldwide, and up to 30% in Eastern Europe. Infertility of unknown etiology is one of the most challenging forms of infertility. Vitamin D has recently been extensively studied and researchers are investigating its possible role in ovulation and pregnancy. While the evidence suggests a positive association between vitamin D and ovulation, more research is needed to understand the role that vitamin D plays in ovulation. (2) Methods: In this study, 86 infertile patients were studied retrospectively in a single center by analyzing their anthropometric (body mass index), biochemical (total cholesterol, insulin resistance) and hormonal data in correlation with their vitamin D status. (3) Results: It was found that the mid-luteal progesterone level was significantly related to the 25-hydroxy vitamin D level in a multivariate linear regression model. An analysis of subgroups of ovulatory and anovulatory patients suggests that serum 25-hydroxy vitamin D levels of approximately 20 ng/mL may be necessary to trigger ovulation or to support progesterone secretion. (4) Conclusions: Vitamin D deficiency should be considered when infertility of unknown etiology and progesterone-related disorders occur.

## 1. Introduction

Infertility is one of today’s health problems affecting a denoted part of the world’s population. The prevalence of fertility problems may reach or even exceed 10–18% among the population [1], but in certain regions, such as Eastern and Central Europe, it can reach 30% [2]. Infertility can be caused by diseases affecting both sexes (20–30%), or it can have a specifically female (50%) or specifically male origin (20–30%) [3]. In addition to primary ovarian insufficiency and space-occupying processes of the uterus and endometriosis, female infertility can also be caused by hormonal changes such as those observed in polycystic ovary syndrome (PCOS) [4].

The relationship between vitamin D (VD) and infertility has recently been a research focus. Studying the role of this seco-steroid in the human body, it turned out that the vitamin D receptor (VDR) is not only found in the organs that regulate calcium homeostasis but also in those that ensure reproduction: in the ovary (mainly in the granulosa cells), uterus, placenta surface, pituitary gland, and hypothalamus [5,6,7,8]. In several studies, low vitamin D levels have been associated with PCOS-specific infertility and insulin resistance (IR) [9,10,11], as well as with low anti-Müllerian hormone levels [12,13,14,15]. Many studies have demonstrated that VD can alter anti-Müllerian hormone signaling, follicle-stimulating hormone (FSH) sensitivity, and progesterone production and release in human granulosa cells, indicating a possible physiologic role for VD in ovarian follicular development and luteinization [14,16]. A recent meta-analysis revealed that vitamin D status influences the ovarian reserve: antral follicle count was significantly lower among Asians, and luteinizing hormone (LH) levels were higher in non-Asians with inadequate vitamin D levels. Furthermore, a negative correlation was found between vitamin D and the LH/FSH ratio in women with normal body mass index [17]. In addition, the active form of VD (1,25-dihydroxy-vitamin D) stimulates aromatase activity [18]. This enzyme converts androgens to estrogens in granulosa cells [19], contributing to a possible imbalance between androgens and estrogens not only in PCOS but also in all low-VD characterized/VD-insufficient women. The results of a meta-analysis published in 2015 clearly showed that low VD levels are more common among women with PCOS than in those without PCOS but did not demonstrate significantly lower levels of vitamin D among women with PCOS compared to non-PCOS controls [20]. Despite this, vitamin D supplementation did not significantly improve the metabolic disorder or the hormonal disorder associated with PCOS [20], which led the authors to conclude that vitamin D deficiency was not the cause but the consequence of the disease. Thus, the role of vitamin D in fertility remains controversial.

There is no consensus regarding the optimal amount of vitamin D to take. Vitamin D supply is best reflected by the long-half-life metabolite 25-hydroxy vitamin D (25-OH-VD), which is produced in large quantities in the liver, but its synthesis might take place in several other tissues, including the brain [21]. In 2011, the Endocrine Society defined a 25-OH-VD blood level between 30 and 100 ng/mL as normal, while its supply is considered insufficient at 20–29.9 ng/mL, deficient below 20 ng/mL, and severely deficient below 15 ng/mL [22]. In Europe, Pludowski et al. consider that the optimal 25-OH-VD concentration for overall health is 30–50 ng/mL (75–125 nmol/L) in the blood, and the range between 20 and 30 ng/mL is suboptimal, below 20 ng/mL deficient, and below 12 ng/mL severely deficient [23,24]. Nevertheless, other biological samples, such as hair, have also been proposed as alternative methods for assessing vitamin D status [25]. The treatment goal is to obtain a minimum level of 20 ng/mL of serum 25-OH-VD in order to reduce the risk of excess mortality, infections, autoimmune diseases, and many other conditions associated with severe vitamin D deficiency [26]. In Europe, according to the HELENA study, 80% of the population has inadequate levels of vitamin D (39.1% with insufficient levels, 27.1% with deficiencies, and 15.1% with severe deficiencies) [27]. According to data from Romanian surveys (between 2012–2014), 1% of the population had vitamin D levels that were too high (most of them infants under one year old), 40% presented adequate levels, and 59% had an insufficient supply (33%) or low vitamin D levels (26%, of which 22% were mild and 4% were severe), which also showed significant seasonal, sex, and age differences [28]. Therefore, an adequate vitamin D level might be critical for reproductive health and function. However, it is unknown whether the “right” VD level is the same for reproductive health and other systems. In infertile women, decreased vitamin D levels might correspond to a higher prevalence of obesity [29]. The risk of endometriosis is also increased when the 25-OH-VD level is deficient or insufficient, but supplementation with high doses of cholecalciferol to achieve 30–40 ng/mL significantly reduced pain [30]. In addition, a strong correlation between hypovitaminosis D and the development of uterine fibroids has been reported [31]. Several studies have reported that vitamin D may play a significant role in inhibiting tumor cell division and reducing tumor size; however, the precise role of this compound and its receptor in the pathophysiology of UFs remains unclear [32]. Conversely, the role of vitamin D levels in artificial reproduction techniques (ART) remains controversial: although some studies found an association of serum and intrafollicular levels of vitamin D with pregnancy rates [33,34], the European Society of Human Reproduction and Embryology (ESHRE) working group did not recommend vitamin D level measurement in ART [35].

In the present study, we examined the vitamin D status of infertile female patients presenting regular menstrual cycles, with the objectives of identifying a possible correlation between the major ovulation biomarker, namely, mid-luteal progesterone level, and the other variables influencing fertility, and determining a minimum level of 25-OH-VD that ensures adequate luteal function.

## 2. Materials and Methods

### 2.1. Participants

In a cross-sectional study covering the period 2014–2019, data from 18- to 45-year-old females diagnosed with infertility from an endocrine outpatient clinic were processed. The inclusion criteria were the presence of ovulation in the last 6 months confirmed by regular menstrual cycles with 3–4 special clinical signs of elevated progesterone level in the luteal phase (fluid retention, breast tenderness, headache, fatigue, sleeping disorders, bloating), or a mid-luteal progesterone level higher than 3 ng/mL [36]. According to the American Society for Reproductive Medicine (ASRM), luteal progesterone values of >3 ng/mL are considered indicative of ovulation [37]. The exclusion criteria were male causes of infertility, diabetes mellitus, gynecological or endocrine causes of reproductive disorders (uterine and adnexal disorders, endometriosis, nonfunctional hyperprolactinaemia and other hypothalamo–hypophyseal disorders, hypo- and hyper-thyroidism, hypogonadisms, PCOS, hyperandrogenisms and hyperandrogenaemia, cortisol secretion disorders, hypercalcemia, genetic syndromes, genital infections, and any kind of medication or restrictive diet). Hirsutism (defined by a modified Ferriman–Gallwey score of 8 or more) and/or acne and/or androgenic alopecia were also considered [38]. Patients with endometriosis, endometrioma, intrauterine fibroids, and polycystic ovarian morphology were excluded based on intravaginal ultrasound criteria or magnetic resonance imaging if they presented with pelvic pain.

The study was carried out with the permission of the Ethics Committee of the George Emil Palade University of Medicine, Pharmacy, Science and Technology of Targu Mures, No. 281/06.12.2018. The study was conducted in compliance with the principles of the Declaration of Helsinki. Accordingly, the data of female patients who did not achieve pregnancy within 12 months of unprotected intercourse in those younger than 35 years, or within 6 months in those older than 35 years were recorded [39]. Considering that menstrual cycle length is not sufficient to diagnose luteal insufficiency, we used the progesterone level to characterize the luteal phase in infertile women who presented regular menstrual cycles (28 ± 7 days). Women presenting menstrual cycle irregularities (shorter than 21 days, longer than 35 days) were not included.

### 2.2. Anthropometric Assessments and Blood Assays

In addition to age, some anthropometric data (such as height and weight) and biochemical parameters (plasma glucose, triglyceride, and total cholesterol levels) were recorded. The hormonal data including thyroid stimulating hormone (TSH), anti-thyroid peroxidase antibody (ATPO), and prolactin (PRL) levels were determined for all of the included patients. In some of the participants, LH, FSH, and total testosterone levels were also measured in the early follicular phase (between the 3rd and 5th days of the menstrual cycle). Blood sampling was performed from each participant after fasting for 10–12 h using standardized sterile tubes. Body mass index (BMI) was calculated as weight in kilograms divided by height in meters squared (kg/m^2^). Fasting glucose, triglyceride, and total cholesterol levels were determined using spectrophotometry. 

An evaluation of the patient’s IR was conducted using the recently proposed triglyceride–glucose (TyG) index, which is based on fasting glucose and triglyceride measurements [40]. The TyG index was considered to be an accurate measure of IR and can outperform the HOMA index, as Vasques et al. demonstrated [41]. 

The TyG index was calculated as follows:

TyG index  =  Ln [fasting triglycerides (mg/dL) × fasting glucose (mg/dL)/2].

In the formula, Ln denotes the natural logarithm.

The serum mid-luteal progesterone, prolactin, and ATPO antibody levels were determined using a chemiluminescent microparticle immunoassay, as well as the LH, FSH, and total testosterone obtained in the early follicular phase. To evaluate the individual’s vitamin D status, serum 25-OH-VD measurement was used. The serum total 25-OH-VD was calculated as the sum of the 25-OH-VD2 and 25-OH-VD3 concentrations, excluding the 3-epi-25-hydroxyvitamin D3 concentration, and expressed as nanograms per milliliter (ng/mL). The results of several studies have shown that different laboratory procedures can produce markedly divergent results regarding the measured 25-OH-VD values for the same sample due both to inter- and intra-assay variability as well as between-laboratory discrepancy. Thus, all 25-OH-VD determinations were carried out by the same laboratory (S.C. Bioclinica SRL, Timisoara, Romania) using the same high-performance liquid chromatographic method. Additionally, to minimize the impact of seasonal variation, only measurements between January and March were taken into account.

### 2.3. Statistical Analysis

The data were processed using GraphPad Prism 9.5 (GraphPad Software Inc., San Diego, CA, USA) as follows: after running the Kolmogorov-Smirnov test, the descriptive statistical results were expressed in the form of mean ± SEM (standard error of means) in the case of normally distributed parameters, and in the form of the median (interquartile range) in the case of non-normally distributed parameters. First, the Spearman rank correlation coefficient was individually calculated only for those variables that were determined in all included patients. Then, multivariate linear regression analysis was used to calculate the correlation between mid-luteal phase progesterone levels and the other covariables: age, BMI, total cholesterol, TyG index, TSH, ATPO antibody, prolactin, and 25-OH-VD levels. A *p* value ≤ 0.05 was considered statistically significant.

Considering the small sample size, a posteriori power data of the statistical analysis were calculated using G* power (version 3.1.9.7) to indicate the statistical power of the outcome variables.

## 3. Results

Eighty-six patients with infertility were included in the study. Their anthropometric and laboratory data are summarized in Table 1. The results of the study showed that except for the progesterone level, the majority of patients had normal hormonal levels and no significant differences in body mass index.

**Figure 1:**
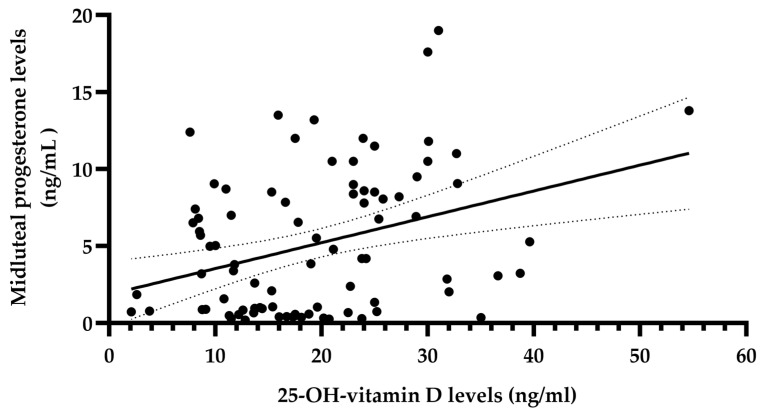
Relationship between serum 25-OH vitamin D levels and mid-luteal progesterone levels in 86 patients with infertility of unknown etiology. The solid line represents the regression line; the dotted lines represent the confidence interval of 95%. Correlation coefficient = 0.28 (*p* < 0.01), 95% CI 0.07–0.47.

The Spearman correlation coefficients (R) calculated between the mid-luteal phase progesterone level and the other variables are presented in Table 2. The correlation coefficients show a significant positive correlation between the mid-luteal phase progesterone level and the serum 25-hydroxy vitamin D concentration (R = 0.280, *p* < 0.01, Figure 1).

A subgroup analysis of the variables was performed between patients showing ovulatory (i.e., mid-luteal progesterone level above 3 ng/mL according to ASMR) and anovulatory cycles. The findings are shown in Table 3. The results showed that there were significant differences between the two groups for only one variable. Notably, the anovulatory group had significantly lower serum 25-hydroxy vitamin D levels than the ovulatory group. All the other variables were similar in both groups.

Based on the assumption that the independent variables are not highly correlated, a multivariate linear regression model was run. Table 4 contains the Variance Inflation Factor (VIF) values. The VIF values indicate the degree of multicollinearity in the model. The higher the value is, the higher the multicollinearity. The VIF values should be less than 10, which has been observed for all the studied variables. The regression model data can then be used to determine which variables have the highest predictive power (Table 5).

Given the small sample size of the present study, Cohen’s d values and posteriori power data were calculated to present the effect sizes and statistical power for the main outcome variables (Table 6). The results showed that the d value/posteriori power for serum 25-hydroxy vitamin D was 0.526/0.773, which presented large effect sizes and statistical power. Therefore, the observed differences in serum 25-hydroxy vitamin D between the sufficient luteal phase and insufficient luteal phase groups are real rather than type II errors.

## 4. Discussion

Vitamin D is a steroid hormone (the active form of the vitamin is 1,25-dihydroxy-vitamin D), with receptors found throughout the body, including in the majority of reproductive organs such as the ovary, the uterus, the placenta, the testis, the hypothalamus, and the pituitary gland itself. Several studies have linked it to a number of pathologies related to reproduction, including endometriosis, PCOS, and infertility.

The physiological effects of vitamin D include the increased production of ovarian steroid hormones, such as progesterone, estrogen and estradiol, and the activation of FSH receptor genes. Vitamin D also increases follicular maturation and selection [42,43]. As a result, there is some evidence that vitamin D enhances ovulation at the ovarian level. In addition, it correlates with anti-Müllerian hormone levels, thus enhancing the sensitivity of FSH [15,44,45].

The mid-luteal progesterone level is frequently used in clinical practice to determine retrospectively whether ovulation has occurred. The minimum ovulation threshold is not well established: according to some authors, it is set between 5 and 8.8 ng/mL [46,47], and according to others, it is above 3 ng/mL [36,37]. It is important to mention that a single determination is not sufficient due to the pulsatile secretion of LH, which controls corpus luteum progesterone secretion. The mid-luteal phase progesterone levels can fluctuate 8-fold during a 90 min period and range from 2.3 to 40.1 ng/mL during a 24 h period in the same healthy person [48]; therefore, a single value can neither diagnose nor exclude ovulation [49]. In the absence of well-defined cutoff limits for serum ovulatory progesterone levels, it is evident that this method has several major drawbacks in defining ovulation. It requires repeated testing, especially when the menstrual cycle is irregular. In this study, three measurements of the serum progesterone were performed and the lowest value was used. The results showed that patients with mid-luteal progesterone levels below 3 ng/mL were associated with low serum levels of 25-hydroxy vitamin D (in general below 20 ng/mL), and a strong positive correlation between serum 25-hydroxy vitamin D and the mid-luteal phase progesterone level was found.

A previous study showed that the active form of vitamin D, calcitriol, increased progesterone synthesis by 13% in cultured human ovarian cells along with estradiol and estrone production [50]. However, a direct link between serum 25-hydroxy vitamin D levels and progesterone synthesis has not yet been established. Recently, Chu et al. studied the correlation between serum total 25-hydroxy vitamin D and several biochemical and endocrinological parameters. The 25-hydroxyvitamin D levels in serum and progesterone levels in the follicular stage of the menstrual cycle did not correlate [51], which does not contradict our results, since progesterone production depends primarily on the corpus luteum’s function.

A recent study of in vitro fertilization patients demonstrated that those with adequate vitamin D levels had a greater chance of obtaining high-quality embryos than those with inadequate vitamin D levels. In addition, women with adequate vitamin D levels were more likely to implant and to become pregnant than those with levels below 20 ng/mL [52]. The preconceptional vitamin D status should therefore be evaluated to increase the chances of conceiving successfully. One study confirmed the role of low vitamin D levels in recurrent miscarriage [53], but no other studies have confirmed the role of vitamin D in recurrent pregnancy loss; hence, the ESHRE does not recommend testing vitamin D status in these cases [54].

Several studies have shown that vitamin D supplementation can improve the clinical pregnancy rate of infertile women. In a recent systematic review and meta-analysis, Meng et al. conducted a subgroup analysis and showed that supplementation with vitamin D only had a limited effect on the clinical pregnancy rate, whereas administering multicomponent products containing vitamin D significantly increased the chance of becoming pregnant. Performing a stratified analysis based on serum vitamin D levels, the authors of the abovementioned meta-analysis claimed that infertile patients with serum vitamin D levels lower than 20 ng/mL obtained no benefit from supplementation, but when the serum vitamin D level was lower than 30 ng/mL before treatment, the clinical pregnancy rate was significantly increased in the case group compared with the control group [55]. While no intervention was implemented and no follow-up studies were conducted in this study, the correlation between serum 25-OH-VD levels and mid-luteal progesterone levels suggests that vitamin D plays a significant role during the luteal phase.

In addition to being beneficial to fertility, vitamin D supplementation may improve birth outcomes and reduce obstetric complications [56,57,58]. There are currently a few randomized control trials on vitamin D supplementation, but its safety, low cost, and potential benefits make it a good recommendation [59,60]. Furthermore, several metabolic disorders associated with infertility, such as PCOS, type 2 diabetes mellitus, high BMI, and IR, have been linked to vitamin D deficiency (for details, see reviews by Mohan et al. [61], Amrein et al. [26], Szymczak-Pajor and Śliwińska [62], and Berry et al. [63]).

Excess body fat is a common feature in infertile women and can contribute to the worsening of the whole clinical picture. Nevertheless, IR may also be found in many normal-weight individuals, and conversely, insulin-sensitive obesity exists [64]. In our study, the IR of patients was characterized, and its correlation with serum 25-OH vitamin D levels was investigated. Currently, there is no specific method for accurately determining IR. Even though they are used in academic studies, euglycemic insulin clamps and intravenous glucose tolerance tests are not being used clinically. The Homeostatic Model Assessment of Insulin Resistance (HOMA-IR), based on fasting glucose and insulin level products, is the most widely used method for IR characterization. A new index for assessing IR in patients with and without diabetes has been developed, the triglyceride–glucose (TyG) index [40,65]. Both fasting triglyceride and fasting glucose measurements are standard components of routine lipid and glucose profiles. Thus, the TyG index can be easily calculated without any additional cost. Compared to HOMA-IR, Vasques et al. mentioned that the TyG index conferred a better performance in identifying patients with IR [41]. The results presented here showed no correlation between the TyG index and mid-luteal progesterone levels, and no difference was observed between ovulatory and anovulatory patients. Notably, the median value of the TyG index in our patients was 8.9 (8.8–9), and in a study of the Spanish population, it was suggested that a TyG value below 8.8 is normal, but a value above 8.9 indicates IR [66]. However, it should be noted that PCOS, according to the Rotterdam criteria [67], was among the exclusion criteria of this study, but the patients were included independent of their BMI.

The limitations of this study include its retrospective and observational nature, that patients were enrolled from a single institution, and that there was no intervention performed. A further limitation could be the relatively small sample size; however, the statistical power of the main outcome can be considered high. Additionally, this study only included infertile women with regular menstrual cycles, so the results may not be applicable to other populations. Ideally, vitamin D status and infertility should be studied in a multicentric setting and in a prospective manner so that confounding factors can be tightly controlled. However, such studies are challenging to conduct due to their long-term nature and high costs. Therefore, we must rely on retrospective studies to gain insights into the relationship between vitamin D status and infertility.

## 5. Conclusions

In conclusion, serum 25-hydroxy vitamin D levels and mid-luteal serum progesterone levels were significantly correlated in our study of a group of females presenting regular menstrual cycles with infertility of unknown origin. This correlation might have implications for fertility and fertility treatments, suggesting the importance of normal vitamin D levels as a prerequisite for a good quality ovulation process. These results might be used to improve pregnancy outcomes and support reproductive health.

## Figures and Tables

**Table 1:** Anthropometric, biochemical, and hormonal characteristics of the patients.

Variable (N = 86)	Mean (±SEM)/Median (Interquartile Range)
Age (years)	31 (±0.66)
BMI (kg/m^2^)	23 (21–26)
Total cholesterol (mg/dL)	159 (147–202)
Triglyceride (mg/dL)	150 (141–171)
Plasma glucose (mg/dL)	93 (89–96)
TyG index	8.9 (8.8–9)
TSH (mIU/L)	1.8 (1–2.8)
ATPO antibody (µIU/mL)	28 (15–48)
Mid-luteal phase progesterone (ng/mL)	4 (0.88–8.5)
Early follicular phase LH (IU/L) *	7.57 (±0.35)
Early follicular phase FSH (IU/L) *	2.2 (1.8–3.1)
Follicular phase total testosterone (ng/mL) *	31 (21–44)
Prolactin (ng/mL)	15 (11–18)
25-OH-VD (ng/mL)	19 (±1)

Normal values: BMI: 18.5–24.9 kg/m^2^, glucose: 72–99 mg%, total cholesterol: 125–200 mg%, triglycerides: 40–150 mg%, TSH: 0.5–4.8 μIU/mL, ATPO: 5–35 IU/L, mid-luteal phase progesterone: 0.5–25 ng/mL, early follicular phase LH and FSH: 2–9 IU/L, early follicular phase total testosterone: 15–90 ng/mL, prolactin: 8–20 ng/mL, 25-OH-VD: 30–100 ng/mL. * Due to missing information, this parameter was not included in statistical analysis (N = 79).

**Table 2:** Correlation between anthropometric and laboratory characteristics and the mid-luteal phase progesterone levels in the study group.

Variable (N = 86)	R	95% CI	*p*
Age (years)	0.021	−0.197 to 0.238	0.846
BMI (kg/m^2^)	−0.131	−0.340 to 0.088	0.225
Total cholesterol (mg/dL)	0.031	−0.187 to 0.247	0.771
TyG index	0.035	−0.184 to 0.251	0.748
TSH (mIU/L)	−0.07	−0.282 to 0.151	0.526
ATPO antibody (µIU/mL)	0.005	−0.212 to 0.223	0.961
Prolactin (ng/mL)	0.116	−0.104 to 0.325	0.287
25-OH-VD (ng/mL)	0.280	0.0668 to 0.469	0.009

**Table 3:** Comparative analysis of data between normal luteal phase and insufficient luteal phase patients.

Variables	Ovulatory Cycles (N = 49)	Anovulatory Cycles (N = 37)	*p*
Age (years)	31.06 ± 0.85	30.54 ± 1.05	0.790 (*t* test)
BMI (kg/m^2^)	22.00 (20.50–25.00)	23.00 (20.50–27.50 d)	0.344 (MW-test)
Total cholesterol (mg/dL)	158 (146.5–204)	160 (147–200)	0.919 (MW test)
TyG index	8.86 (8.77–8.95)	8.82 (8.73–8.99)	0.619 (MW test)
TSH (mIU/L)	1.89 ± 0.14	2.07 ± 0.16	0.423 (*t* test)
ATPO antibody (µIU/mL)	22.9 (20.9–25.3)	25 (15–45)	0.730 (MW-test)
Prolactin (ng/mL)	14.79 ± 0.64	13.68 ± 0.71	0.251 (*t* test)
25-OH-VD (ng/mL)	21.38 ± 1.44	16.70 ± 1.23	0.020 (*t* test)

Data are expressed as mean (±SEM)/median (interquartile range) depending on the distribution of the dataset. MW: Mann–Whitney test.

**Table 4:** Variance Inflation Factor values of the independent variables.

Variables	VIF	R^2^ with Other Variables
Age (years)	1.078	0.0719
BMI (kg/m^2^)	1.150	0.1305
Total cholesterol (mg/dL)	1.103	0.0937
TyG index	1.106	0.0954
TSH (mIU/L)	1.054	0.0508
ATPO antibody (µIU/mL)	1.074	0.0686
Prolactin (ng/mL)	1.071	0.0663
25-OH-VD (ng/mL)	1.085	0.0781

**Table 5:** The linear regression model data when the outcome variable was the mid-luteal phase progesterone level.

Variables	Estimate	SE	95% CI	|t|	*p*
Age (years)	0.031	0.080	−0.129 to 0.191	0.385	0.700
BMI (kg/m^2^)	−0.069	0.102	−0.273 to 0.133	0.681	0.497
Total cholesterol (mg/dL)	0.016	0.014	−0.011 to 0.044	1.173	0.244
TyG index	−0.138	2.406	−4.929 to 4.654	0.057	0.954
TSH (mIU/L)	−0.591	0.493	−1.574 to 0.390	1.200	0.233
ATPO antibody (µIU/mL)	0.001	0.003	−0.005 to 0.008	0.438	0.662
Prolactin (ng/mL)	0.056	0.111	−0.165 to 0.278	0.504	0.615
25-OH-VD (ng/mL)	0.173	0.053	0.067 to 0.278	3.259	0.001

Notes: Degrees of Freedom 77, Multiple R 0.406, R squared 0.165, Adjusted R squared 0.078.

**Table 6:** Differences in variables between the patients presenting anovulatory cycles (N = 37) and ovulatory cycles (N = 49) subgroups.

Variables	Δ (Means/Medians) ± SEM	Effect Size (d)	*A* Posteriori Power Data
Age (years)	−0.5207 ± 1.341	0.084	0.103
BMI (kg/m^2^)	−1.00	0.268	0.229
Total cholesterol (mg/dL)	2.00	0.020	0.060
TyG index	0.049	0.005	0.052
TSH (mIU/L)	0.1738 ± 0.2158	0.175	0.199
ATPO antibody (µIU/mL)	0.00	0.434	0.486
Prolactin (ng/mL)	−1.111 ± 0.9600	0.252	0.208
25-OH-VD (ng/mL)	−4.680 ± 1.978	0.526	0.773

Δ are expressed as differences of the means (±SEM)/median depending on the distribution of the dataset.

## Data Availability

The data presented in this study are available on request from the corresponding author.

## References

[1] Aghajanova L., Hoffman J., Mok-Lin E., Herndon C.N. (2017). Obstetrics and Gynecology Residency and Fertility Needs: National Survey Results. Reprod. Sci..

[2] Vander Borght M., Wyns C. (2018). Fertility and infertility: Definition and epidemiology. Clin. Biochem..

[3] Agarwal A., Mulgund A., Hamada A., Chyatte M.R. (2015). A unique view on male infertility around the globe. Reprod. Biol. Endocrinol..

[4] Hanson B., Johnstone E., Dorais J., Silver B., Peterson C.M., Hotaling J. (2017). Female infertility, infertility-associated diagnoses, and comorbidities: A review. J. Assist. Reprod. Genet..

[5] Irani M., Merhi Z. (2014). Role of vitamin D in ovarian physiology and its implication in reproduction: A systematic review. Fertil. Steril..

[6] Xu J., Lawson M.S., Xu F., Du Y., Tkachenko O.Y., Bishop C.V., Pejovic-Nezhat L., Seifer D.B., Hennebold J.D. (2018). Vitamin D3 Regulates Follicular Development and Intrafollicular Vitamin D Biosynthesis and Signaling in the Primate Ovary. Front. Physiol..

[7] Muscogiuri G., Altieri B., de Angelis C., Palomba S., Pivonello R., Colao A., Orio F. (2017). Shedding new light on female fertility: The role of vitamin D. Rev. Endocr. Metab. Disord..

[8] Wehr E., Trummer O., Giuliani A., Gruber H.-J., Pieber T.R., Obermayer-Pietsch B. (2011). Vitamin D-associated polymorphisms are related to insulin resistance and vitamin D deficiency in polycystic ovary syndrome. Eur. J. Endocrinol..

[9] Guo S., Tal R., Jiang H., Yuan T., Liu Y. (2020). Vitamin D Supplementation Ameliorates Metabolic Dysfunction in Patients with PCOS: A SystematicReview of RCTs and Insight into the Underlying Mechanism. Int. J. Endocrinol..

[10] Morgante G., Darino I., Spanò A., Luisi S., Luddi A., Piomboni P., Governini L., De Leo V. (2022). PCOS Physiopathology and Vitamin D Deficiency: Biological Insights and Perspectives for Treatment. J. Clin. Med..

[11] Łagowska K., Bajerska J., Jamka M. (2018). The Role of Vitamin D Oral Supplementation in Insulin Resistance in Women with Polycystic Ovary Syndrome: A Systematic Review and Meta-Analysis of Randomized Controlled Trials. Nutrients.

[12] Grineva E., Karonova T., Micheeva E., Belyaeva O., Belyaeva I. (2013). Vitamin D deficiency is a risk factor for obesity and diabetes type 2 in women at late reproductive age. Aging.

[13] Krul-Poel Y.H.M., Koenders P.P., Steegers-Theunissen R.P., ten Boekel E., ter Wee M.M., Louwers Y., Lips P., Laven J.S.E., Simsek S. (2018). Vitamin D and metabolic disturbances in polycystic ovary syndrome (PCOS): A cross-sectional study. PLoS ONE.

[14] Dennis N., Houghton L., Pankhurst M., Harper M., McLennan I. (2017). Acute Supplementation with High Dose Vitamin D3 Increases Serum Anti-Müllerian Hormone in Young Women. Nutrients.

[15] Moridi I., Chen A., Tal O., Tal R. (2020). The Association between Vitamin D and Anti-Müllerian Hormone: A Systematic Review and Meta-Analysis. Nutrients.

[16] Jukic A.M.Z., Baird D.D., Wilcox A.J., Weinberg C.R., Steiner A.Z. (2018). 25-Hydroxyvitamin D (25(OH)D) and biomarkers of ovarian reserve. Menopause.

[17] Karimi E., Arab A., Rafiee M., Amani R. (2021). A systematic review and meta-analysis of the association between vitamin D and ovarian reserve. Sci. Rep..

[18] Lou Y.-R., Murtola T., Tuohimaa P. (2005). Regulation of aromatase and 5α-reductase by 25-hydroxyvitamin D3, 1α,25-dihydroxyvitamin D3, dexamethasone and progesterone in prostate cancer cells. J. Steroid Biochem. Mol. Biol..

[19] Mahmoudi T., Gourabi H., Ashrafi M., Yazdi R.S., Ezabadi Z. (2010). Calciotropic hormones, insulin resistance, and the polycystic ovary syndrome. Fertil. Steril..

[20] He C., Lin Z., Robb S., Ezeamama A. (2015). Serum Vitamin D Levels and Polycystic Ovary syndrome: A Systematic Review and Meta-Analysis. Nutrients.

[21] Gáll Z., Székely O. (2021). Role of vitamin d in cognitive dysfunction: New molecular concepts and discrepancies between animal and human findings. Nutrients.

[22] Holick M.F., Binkley N.C., Bischoff-Ferrari H.A., Gordon C.M., Hanley D.A., Heaney R.P., Murad M.H., Weaver C.M. (2011). Evaluation, Treatment, and Prevention of Vitamin D Deficiency: An Endocrine Society Clinical Practice Guideline. J. Clin. Endocrinol. Metab..

[23] Pludowski P., Holick M.F., Grant W.B., Konstantynowicz J., Mascarenhas M.R., Haq A., Povoroznyuk V., Balatska N., Barbosa A.P., Karonova T. (2018). Vitamin D supplementation guidelines. J. Steroid Biochem. Mol. Biol..

[24] Płudowski P., Kos-Kudła B., Walczak M., Fal A., Zozulińska-Ziółkiewicz D., Sieroszewski P., Peregud-Pogorzelski J., Lauterbach R., Targowski T., Lewiński A. (2023). Guidelines for Preventing and Treating Vitamin D Deficiency: A 2023 Update in Poland. Nutrients.

[25] Gáll Z., Csukor B., Urkon M., Farczádi L., Kolcsár M. (2022). Vitamin D Status Assessment: Lack of Correlation between Serum and Hair 25-Hydroxycholecalciferol Levels in Healthy Young Adults. Diagnostics.

[26] Amrein K., Scherkl M., Hoffmann M., Neuwersch-Sommeregger S., Köstenberger M., Tmava Berisha A., Martucci G., Pilz S., Malle O. (2020). Vitamin D deficiency 2.0: An update on the current status worldwide. Eur. J. Clin. Nutr..

[27] González-Gross M., Valtueña J., Breidenassel C., Moreno L.A., Ferrari M., Kersting M., De Henauw S., Gottrand F., Azzini E., Widhalm K. (2012). Vitamin D status among adolescents in Europe: The Healthy Lifestyle in Europe by Nutrition in Adolescence study. Br. J. Nutr..

[28] Chirita-Emandi A., Socolov D., Haivas C., Calapiș A., Gheorghiu C., Puiu M. (2015). Vitamin D Status: A Different Story in the Very Young versus the Very Old Romanian Patients. PLoS ONE.

[29] Zhao J., Fu S., Chen Q. (2023). Association between the serum vitamin D level and prevalence of obesity/abdominal obesity in women with infertility: A cross-sectional study of the National Health and Nutrition Examination Survey data. Gynecol. Endocrinol..

[30] Kalaitzopoulos D.R., Samartzis N., Daniilidis A., Leeners B., Makieva S., Nirgianakis K., Dedes I., Metzler J.M., Imesch P., Lempesis I.G. (2022). Effects of vitamin D supplementation in endometriosis: A systematic review. Reprod. Biol. Endocrinol..

[31] Ciebiera M., Włodarczyk M., Ciebiera M., Zaręba K., Łukaszuk K., Jakiel G. (2018). Vitamin D and Uterine Fibroids—Review of the Literature and Novel Concepts. Int. J. Mol. Sci..

[32] Guo W., Dai M., Zhong Z., Zhu S., Gong G., Chen M., Guo J., Zhang Y. (2022). The association between vitamin D and uterine fibroids: A mendelian randomization study. Front. Genet..

[33] Ozkan S., Jindal S., Greenseid K., Shu J., Zeitlian G., Hickmon C., Pal L. (2010). Replete vitamin D stores predict reproductive success following in vitro fertilization. Fertil. Steril..

[34] Baldini D., Malvasi A., Kosmas I., Baldini G.M., Silvestris E. (2021). Increased bioavailability of Vitamin D improved pregnancy outcomes in in vitro fertilization cycles, only in patients over 36 years: A cross-sectional study. Eur. Rev. Med. Pharmacol. Sci..

[35] Cimadomo D., de los Santos M.J., Griesinger G., Lainas G., Le Clef N., McLernon D.J., Montjean D., Toth B., Vermeulen N., Macklon N. (2023). ESHRE good practice recommendations on recurrent implantation failure. Hum. Reprod. Open.

[36] Practice Committee of the American Society for Reproductive Medicine (2015). Diagnostic evaluation of the infertile female: A committee opinion. Fertil. Steril..

[37] Practice Committees of the American Society for Reproductive Medicine and the Society for Reproductive Endocrinology and Infertility (2021). Diagnosis and treatment of luteal phase deficiency: A committee opinion. Fertil. Steril..

[38] Aswini R., Jayapalan S. (2017). Modified Ferriman–Gallwey score in hirsutism and its association with metabolic syndrome. Int. J. Trichology.

[39] Committee on Gynecologic Practice, American Society for Reproductive Medicine (2019). Infertility Workup for the Women’s Health Specialist: ACOG Committee Opinion, Number 781. Obstet. Gynecol..

[40] Simental-Mendía L.E., Rodríguez-Morán M., Guerrero-Romero F. (2008). The Product of Fasting Glucose and Triglycerides as Surrogate for Identifying Insulin Resistance in Apparently Healthy Subjects. Metab. Syndr. Relat. Disord..

[41] Vasques A.C.J., Novaes F.S., de Oliveira M.d.S., Matos Souza J.R., Yamanaka A., Pareja J.C., Tambascia M.A., Saad M.J.A., Geloneze B. (2011). TyG index performs better than HOMA in a Brazilian population: A hyperglycemic clamp validated study. Diabetes Res. Clin. Pract..

[42] Voulgaris N., Papanastasiou L., Piaditis G., Angelousi A., Kaltsas G., Mastorakos G., Kassi E. (2017). Vitamin D and aspects of female fertility. Hormones.

[43] Várbíró S., Takács I., Tűű L., Nas K., Sziva R.E., Hetthéssy J.R., Török M. (2022). Effects of Vitamin D on Fertility, Pregnancy and Polycystic Ovary Syndrome—A Review. Nutrients.

[44] Bednarska-Czerwińska A., Olszak-Wąsik K., Olejek A., Czerwiński M., Tukiendorf A. (2019). Vitamin D and Anti-Müllerian Hormone Levels in Infertility Treatment: The Change-Point Problem. Nutrients.

[45] Grzeczka A., Graczyk S., Skowronska A., Skowronski M.T., Kordowitzki P. (2022). Relevance of Vitamin D and Its Deficiency for the Ovarian Follicle and the Oocyte: An Update. Nutrients.

[46] Wathen N.C., Perry L., Lilford R.J., Chard T. (1984). Interpretation of single progesterone measurement in diagnosis of anovulation and defective luteal phase: Observations on analysis of the normal range. BMJ.

[47] Leiva R., Bouchard T., Boehringer H., Abulla S., Ecochard R. (2015). Random serum progesterone threshold to confirm ovulation. Steroids.

[48] Filicori M., Butler J.P., Crowley W.F. (1984). Neuroendocrine regulation of the corpus luteum in the human. Evidence for pulsatile progesterone secretion. J. Clin. Investig..

[49] Mesen T.B., Young S.L. (2015). Progesterone and the Luteal Phase. Obstet. Gynecol. Clin. N. Am..

[50] Parikh G., Varadinova M., Suwandhi P., Araki T., Rosenwaks Z., Poretsky L., Seto-Young D. (2010). Vitamin D Regulates Steroidogenesis and Insulin-like Growth Factor Binding Protein-1 (IGFBP-1) Production in Human Ovarian Cells. Horm. Metab. Res..

[51] Chu C., Tsuprykov O., Chen X., Elitok S., Krämer B.K., Hocher B. (2021). Relationship Between Vitamin D and Hormones Important for Human Fertility in Reproductive-Aged Women. Front. Endocrinol..

[52] Chu J., Gallos I., Tobias A., Robinson L., Kirkman-Brown J., Dhillon-Smith R., Harb H., Eapen A., Rajkhowa M., Coomarasamy A. (2019). Vitamin D and assisted reproductive treatment outcome: A prospective cohort study. Reprod. Health.

[53] Ota K., Dambaeva S., Han A.-R., Beaman K., Gilman-Sachs A., Kwak-Kim J. (2014). Vitamin D deficiency may be a risk factor for recurrent pregnancy losses by increasing cellular immunity and autoimmunity. Hum. Reprod..

[54] Bender Atik R., Christiansen O.B., Elson J., Kolte A.M., Lewis S., Middeldorp S., Mcheik S., Peramo B., Quenby S., Nielsen H.S. (2022). ESHRE guideline: Recurrent pregnancy loss: An update in 2022. Hum. Reprod. Open.

[55] Meng X., Zhang J., Wan Q., Huang J., Han T., Qu T., Yu L. (2023). Influence of Vitamin D supplementation on reproductive outcomes of infertile patients: A systematic review and meta-analysis. Reprod. Biol. Endocrinol..

[56] Sablok A., Batra A., Thariani K., Batra A., Bharti R., Aggarwal A.R., Kabi B.C., Chellani H. (2015). Supplementation of vitamin D in pregnancy and its correlation with feto-maternal outcome. Clin. Endocrinol..

[57] Rostami M., Tehrani F.R., Simbar M., Bidhendi Yarandi R., Minooee S., Hollis B.W., Hosseinpanah F. (2018). Effectiveness of Prenatal Vitamin D Deficiency Screening and Treatment Program: A Stratified Randomized Field Trial. J. Clin. Endocrinol. Metab..

[58] Mumford S.L., Garbose R.A., Kim K., Kissell K., Kuhr D.L., Omosigho U.R., Perkins N.J., Galai N., Silver R.M., Sjaarda L.A. (2018). Association of preconception serum 25-hydroxyvitamin D concentrations with livebirth and pregnancy loss: A prospective cohort study. Lancet Diabetes Endocrinol..

[59] Bialy L., Fenton T., Shulhan-Kilroy J., Johnson D.W., McNeil D.A., Hartling L. (2020). Vitamin D supplementation to improve pregnancy and perinatal outcomes: An overview of 42 systematic reviews. BMJ Open.

[60] Cavoretto P.I., Viganò P. (2022). Time to implement vitamin D assessment and supplementation into routine obstetric practice?. Fertil. Steril..

[61] Mohan A., Haider R., Fakhor H., Hina F., Kumar V., Jawed A., Majumder K., Ayaz A., Lal P.M., Tejwaney U. (2023). Vitamin D and polycystic ovary syndrome (PCOS): A review. Ann. Med. Surg..

[62] Szymczak-Pajor I., Śliwińska A. (2019). Analysis of Association between Vitamin D Deficiency and Insulin Resistance. Nutrients.

[63] Berry S., Seidler K., Neil J. (2022). Vitamin D deficiency and female infertility: A mechanism review examining the role of vitamin D in ovulatory dysfunction as a symptom of polycystic ovary syndrome. J. Reprod. Immunol..

[64] Lee S., Lacy M.E., Jankowich M., Correa A., Wu W.-C. (2020). Association between obesity phenotypes of insulin resistance and risk of type 2 diabetes in African Americans: The Jackson Heart Study. J. Clin. Transl. Endocrinol..

[65] Placzkowska S., Pawlik-Sobecka L., Kokot I., Piwowar A. (2019). Indirect insulin resistance detection: Current clinical trends and laboratory limitations. Biomed. Pap..

[66] Sánchez-Íñigo L., Navarro-González D., Fernández-Montero A., Pastrana-Delgado J., Martínez J.A. (2016). The TyG index may predict the development of cardiovascular events. Eur. J. Clin. Investig..

[67] Rotterdam ESHRE/ASRM-Sponsored PCOS Consensus Workshop Group (2004). Revised 2003 consensus on diagnostic criteria and long-term health risks related to polycystic ovary syndrome (PCOS). Hum. Reprod..

